# *In Vitro* Anti-*Listerial* Activities of Crude *n-*Hexane and Aqueous Extracts of *Garcinia kola* (heckel) Seeds

**DOI:** 10.3390/ijms12106952

**Published:** 2011-10-19

**Authors:** Dambudzo Penduka, Anthony I. Okoh

**Affiliations:** Applied and Environmental Microbiology Research Group (AEMREG), Department of Biochemistry and Microbiology, University of Fort Hare, Alice, South Africa; E-Mail: aokoh@ufh.ac.za

**Keywords:** *Garcinia kola* seeds, *Listeria* species, rate of kill, bactericidal

## Abstract

We assessed the anti-*Listerial* activities of crude n-hexane and aqueous extracts of *Garcinia kola* seeds against a panel of 42 *Listeria* isolates previously isolated from wastewater effluents in the Eastern Cape Province of South Africa and belonging to *Listeria monocytogenes*, *Listeria grayi* and *Listeria ivanovii* species. The *n*-hexane fraction was active against 45% of the test bacteria with zones of inhibition ranging between 8–17 mm, while the aqueous fraction was active against 29% with zones of inhibition ranging between 8–11 mm. The minimum inhibitory concentrations (MIC) were within the ranges of 0.079–0.625 mg/mL for the *n*-hexane extract and 10 to >10 mg/mL for the aqueous extract. The rate of kill experiment carried out for the *n*-hexane extract only, revealed complete elimination of the initial bacterial population for *L. grayi* (LAL 15) at 3× and 4× MIC after 90 and 60 min; *L. monocytogenes* (LAL 8) at 3× and 4× MIC after 60 and 15 min; *L. ivanovii* (LEL 18) at 3× and 4× MIC after 120 and 15 min; *L. ivanovii* (LEL 30) at 2, 3 and 4× MIC values after 105, 90 and 15 min exposure time respectively. The rate of kill activities were time- and concentration-dependant and the extract proved to be bactericidal as it achieved a more than 3log_10_ decrease in viable cell counts after 2 h exposure time for all of the four test organisms at 3× and 4× MIC values. The results therefore show the potential presence of anti-*Listerial* compounds in *Garcinia kola* seeds that can be exploited in effective anti-*Listerial* chemotherapy.

## 1. Introduction

The genus *Listeria* consists of Gram-positive, non-sporeforming rod shaped bacteria which are facultatively anaerobic, catalase positive, oxidase negative and ubiquitous in nature [1]. There are six characterized *Listeria* species, namely *Listeria monocytogenes*, *Listeria ivanovii*, *Listeria seeligeri*, *Listeria innocua*, *Listeria grayi* and *Listeria welshimeri* [1,2]. Among these six species only two species *L. ivanovii* and *L. monocytogenes* are regarded as pathogenic; *L. monocytogenes* is pathogenic to humans and animals whilst *L. ivanovii* is pathogenic to animals mainly sheep and cattle causing the bacteremia listeriosis [2,3]. Some studies have however implicated *L. seeligeri* [4], *L. grayi* [1,5,6] and *L. ivanovii* in human listeriosis [7,8] and *L. innocua* in animal listeriosis [9] thereby also showing the potential pathogenicity of some of the *Listeria* species apart from *L. monocytogenes* and *L. ivanovii*.

Listeriosis is a severe food-borne disease characterized by bacteremia, meningitis and encephalitis individuals usually at high risk are those with impaired cell-mediated immunity, including neonates, pregnant woman, elderly persons, and the immune-compromised patients [10]. *L. monocytogenes* is an invasive, intracellular pathogen that can transverse the placenta in pregnant women and infect the fetus, although some intrauterine infection may be the result of ascending spread of the bacteria from vaginal colonization resulting in abortion, birth of a stillborn fetus or a baby with generalized infection (granulomatosis infantiseptica), and sepsis or meningitis in the neonate such that it is of particular high risk for pregnant women [11–13].

Listeriosis is regarded as a food-borne disease because most of the listeriosis cases are mainly caused by consumption of contaminated food [2,13,14], foods such as ready-to-eat meat products and milk products such as cheese [13,15]. In addition to its ubiquitous nature, the *Listeria* species presents a particular concern with respect to food handling because of the ability to grow at temperatures of 0–45 °C making the species able to grow at refrigerator temperatures commonly used to control pathogens in foods. It can also multiply at high salt concentration (10% Sodium chloride) and at pH values ranging 4.5–9 [2,16].

Standard antibiotic therapy for the effective treatment of listeriosis consists of the intravenous administration of penicillin or ampicillin often in combination with an aminoglycoside. The drug of choice in patients with a known allergy to penicillins is vancomycin/teicoplanin or trimethoprim/sulfamethoxazole [15]. Listeriosis has an average case-fatality rate of 20–30% despite adequate antibiotic treatment [15] and case fatality rates as high as 40% have been reported during outbreaks [11,17]. Epidemiological surveillances have shown the prevalence of antibiotic resistant strains of *Listeria* species to different antibiotics including those used for the treatment of listeriosis [12,18–20].

The challenge is therefore to develop effective strategies that may be able to help curb antibiotic resistance in such virulent bacteria species such as *Listeria*. Traditional medicinal plants such as *Garcinia kola* which is a plant of Central and West African origin have been shown to be potential sources of anti-bacterial compounds that can be effective against antibiotic resistant bacteria species. *Garcinia kola* is an evergreen, well branched medium-sized tree growing up to 12 metres tall and 1.5 metres wide in 12 years. It has a regular fruiting cycle and produces a characteristic orange-like pod, with edible portion contained in the pod yearly and it belongs to the family *Guttiferae* [21–23]. *Garcinia kola* seed also known as “bitter kola” because of its bitter taste has been and is still used traditionally to treat various medicinal ailments such as diarrhoea, hepatitis, asthma, dysmenorrhea, diabetes, anaemia, angina, liver disorders and also as an antidote against ingested poison [24,25].

Adedeji *et al*. [22] studies showed that the inclusion of *Garcinia kola* seed powder into the diet of pullet chickens lowered their mortality rate and also caused significant proliferation of the chickens’ white blood cells specifically the lymphocytes [22]. Lymphocytes play an important role in cellular immunity as they form antibodies that attack antigens in the body; this further supports the traditional medicinal value of *Garcinia kola* seeds. There have also been studies by various authors that have also proven the antimicrobial activities of the seeds of this plant [26–30]. In studies by Han *et al*. [31] an antibacterial biflavonoid 3″,4′,4‴,5,5″,7,7″-heptahydroxy-3,8″-biflavanone (GB1) was isolated from the roots of *Garcinia kola* and the GB1 showed antibacterial activities against methicillin-resistant *Staphylococcus aureus* (MRSA) and vancomycin-resistant enterococci (VRE) [31].

The vast medicinal properties and therapeutic potentials of *Garcinia kola* seeds and the high listeriosis mortality rates in particular against a background of prevalent antibiotic resistant *Listeria* species, prompted this study to evaluate the anti-*Listerial* properties of the *Garcinia kola* seed. Despite the numerous studies that have been done on this plant, to the best of our knowledge there is no information in the literature on the *in vitro* anti-*listerial* activities of *n*-hexane and aqueous extracts of the *Garcinia kola* seed.

## 2. Results and Discussion

### 2.1. Results

The results of the anti-*Listerial* activities of the crude extracts are shown in Table 1. The *n*-hexane extract had activity against 19 isolates whilst the aqueous extract had activity against 12 isolates in total with all the isolates that were susceptible to the aqueous extract also being susceptible to the *n*hexane extract. The zones of inhibition ranged from 8–17 mm and 8–11 mm for the *n*-hexane and aqueous extracts respectively at a concentration of 10 mg/mL. The highest zone of inhibition for the *n*hexane extract was 17 mm obtained against *L. ivanovii* (LEL30) and *L. ivanovii* (LDB 7) whilst for the aqueous extract it was 11 mm obtained against *L. ivanovii* (LEL 1). The positive control (Ciprofloxacin) and negative control (5% DMSO) were used for quality control purposes, with the positive control showing activity against all the isolates with inhibition zones ranging from 9–35 mm whilst, the negative control had no activity against all the isolates.

Table 2 shows the MIC and MBC results for both the extracts against the susceptible *Listeria* isolates. The *n*-hexane extract had MIC values ranges of 0.079–0.625 mg/mL with a mean value of 0.218 mg/mL, whilst the MBC values ranges were 0.625–10 mg/mL with a mean value of 8.717 mg/mL. The aqueous extract had MIC values between 10 to >10 mg/mL and MBC values above 10 mg/mL for all the isolates. The *n*-hexane interms of its lower MIC and MBC values proved to be more active in comparison to the aqueous extract.

**Figure 1:**
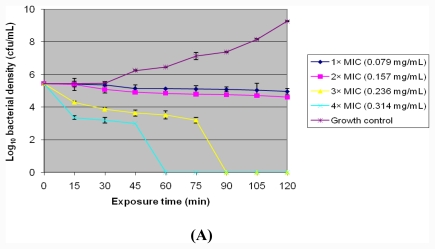

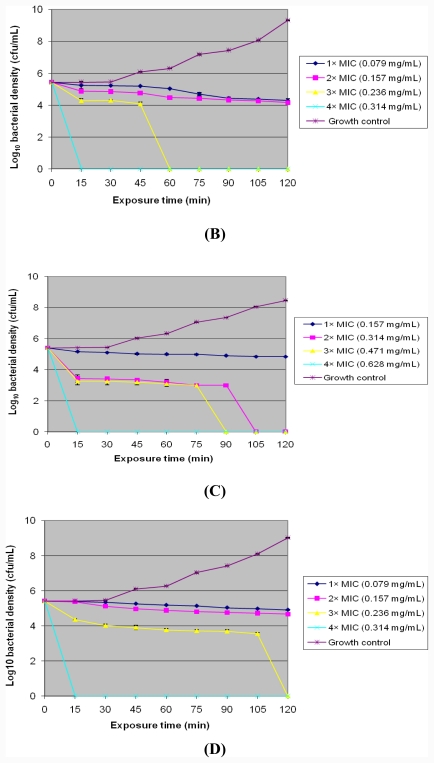
(A) Profile of rate of kill of *L. grayi* (LAL 15) by crude *n*-hexane extracts of *Garcinia kola* seeds; (B) Profile of rate of kill of *L. monocytogenes* (LAL 8) by crude *n*hexane extracts of *Garcinia kola* seeds; (C) Profile of rate of kill of *L. ivanovii* (LEL 30) by crude *n*-hexane extracts of *Garcinia kola* seeds; (D) Profile of rate of kill of *L. ivanovii* (LEL 18) by crude *n*-hexane extracts of *Garcinia kola* seeds.

The results for the rate of kill assay for the *n*-hexane extract against the four representative *Listeria* species are shown in Figures 1(A),1(B),1(C) and 1(D) with standard deviations included in the curves for *L. grayi* (LAL 15), *L. monocytogenes* (LAL 8), *L. ivanovii* (LEL 30) and *L. ivanovii* (LEL 18) respectively. The rate of kill proved to be both time- and concentration-dependent for all the organisms. A complete bactericidal effect for *L. grayi* (LAL 15) was achieved at both 3× MIC and 4× MIC after 90 and 60 min exposure time. *L. monocytogenes*’ (LAL 8) entire bacterial population was wiped out at both 3× MIC and 4× MIC after 60 and 15 min exposure time respectively. *L. ivanovii’s* (LEL 30) entire bacterial population was eliminated at 2, 3 and 4× MIC values after 105, 90 and 15 min respectively and a complete bactericidal effect for *L. ivanovii* (LEL 18) was achieved at 3× MIC and 4× MIC values after 120 and 15 min exposure time respectively. The *n*-hexane extract proved to be bactericidal against all the *Listeria* species giving a more than 3log_10_ decrease in viable cell counts after 2 h exposure time.

### 2.2. Discussion

The crude *n*-hexane and aqueous extracts of *Garcinia kola* seeds showed appreciable anti-*Listerial* activities from the susceptibility tests results with the *n*-hexane extract achieving a 45% activity which was higher in comparison to a 29% activity of the aqueous extract. The MIC and MBC ranges of the *n*-hexane extract were ranging between 0.079–0.625 mg/mL and 0.625–10 mg/mL respectively whilst the aqueous extract had higher values with MIC and MBC ranges of 10 to >10 mg/mL and above 10 mg/mL respectively. These results corroborates other reports [28,32–35] that showed that the organic solvents extracts of *Garcinia kola* seeds are more antibacterial in comparison to the aqueous extracts, mainly because of the better solubility of the antibacterial agents in *Garcinia kola* such as xanthones, benzophenones, and flavonoids [31,36,37] in organic solvents than in water [29,34,38–40].

Some studies on the antibacterial activities of the crude aqueous extracts of *Garcinia kola* seeds have shown MIC values within the ranges of 5–20 mg/mL [28,29,33]. Similarly, in our findings, 10 isolates had MIC values of 10 mg/mL whilst only two had MIC values above 10 mg/mL. Variations in the methodologies used in the studies becomes the greatest obstacle in comparing results to give concrete evidence of the seeds’ crude aqueous extracts MIC ranges but they however support the use of the seeds’ aqueous extracts in traditional medicine to treat various medical conditions that can originate from bacterial infections such as diarrhoea, high fever and throat infections [21].

Members of the genus *Garcinia* from the family Guitefferae are considered as a rich and valuable source of bioactive compounds [41–44]. In a study by Pereira *et al*. [43], three prenylated benzophenones namely 7-epi-clusianone, garciniaphenone and guttiferone-a were obtained from silica gel chromatography of the hexane extract of powdered *Garcinia brasiliensis* Mart. fruits and these were found to exhibit significant activity on *Leishmania* (*L.*) *amazonensis* and having minimum toxicity for mammalian cells [43]. In a separate study involving non-polar solvents petroleum ether and ethyl acetate a polyisoprenyl benzophenone (kolanone) was found in the petroleum ether fraction whilst a hydroxybiflavanonols was found in the ethyl acetate fraction of *Garcinia kola* seeds and GB1 (a hydroxybiflavanonol) was the main component exhibiting activity against bacteria, *Candida albicans* and *Aspergillus flavus* [45]. The activity of the *n*-hexane extract in this study can therefore be attributed to a number of compounds possibly those mentioned above that can be found in *Garcinia kola* seeds especially those extracted through the use of non-polar solvents such as *n*-hexane.

Rate of kill assays show the bactericidal activity or the duration of a bacteriostatic effect of a fixed concentration of the antimicrobial agent, thereby providing a clear analysis of the relationship between the extent of microbial population mortality and the antimicrobial agent concentration [46,47].The rate of kill activity of the *n*-hexane extract proved to be bactericidal at ×2 (for *L. ivanovii* (LEL 30) only), ×3 and ×4 MIC values after 2 h exposure time for all the test organisms, since a reduction of the viable bacterial density of ≥99.9% or ≥3log_10_ in cfu/mL is used as a standard of measurement for bactericidal efficacy [48,49]. The acetone extracts [33], methanol extracts [29], butanol and diethyl-ether fractions of the methanol extract [50] of *Garcinia kola* seeds were also found to exhibit bactericidal activities against both Gram positive and Gram negative bacteria, with the findings of Akinpelu *et al*.[50] and Sibanda and Okoh [33] showing a concentration- and time-dependent killing activity similar to this study.

This study therefore shows the nature of inhibition of the *n*-hexane extract of *Garcinia kola* seeds to be bactericidal at 3× and 4× MIC values against *Listeria* species as well as being concentration-and time-dependent.

## 3. Materials and Methods

### 3.1. Plant Material

*Garcinia kola* seeds ground powder was obtained from the plant material collection of the Applied and Environmental Microbiology Research Group (AEMREG) laboratory, University of Fort Hare Alice, South Africa.

### 3.2. Preparation of Extracts

The method of Basri and Fan [51] was used to prepare the solvents extracts. The seed powder (100 grams) was steeped in 500 mL of the respective solvent (*n*-hexane or water) for 48 h with shaking in an orbital shaker (Stuart Scientific Orbital Shaker, UK). The resultant extract was centrifuged at 3000 rpm for 5 min at 4 °C (Beckman Model TJ-6RS Centrifuge, Great Britain), the supernatant was then filtered through Whatman No.1 filter paper while the residue was then used in the second extraction process with 300 mL of the respective solvents. After which the combined aqueous extract was freeze-dried at −50 °C under vacuum, whereas the *n*-hexane extracts were concentrated under reduced pressure using a rotary evaporator at 50 °C. The concentrated extracts were then allowed to dry to a constant weight under a stream of air in a fume cupboard at room temperature. Dimethyl sulphoxide (DMSO) at a concentration equal to 5% of the total volume which was made up with sterile distilled water was used to aid the reconstitution of the dried *n*-hexane extract when making different test concentrations whilst the water extracts were reconstituted in sterile distilled water.

### 3.3. Test *Listeria* Strains

The test *Listeria* isolates (42 in all) used in this study were obtained from the culture collection of the Applied and Environmental Microbiology Research Group (AEMREG) laboratory at the University of Fort Hare, Alice, South Africa. The bacteria were previously isolated from wastewater effluents and belonged to three specie groups which are *L. ivanovii*, *L. grayi* and *L. monocytogenes* [20].

### 3.4. Preparation of the Inoculum

The colony suspension method according to EUCAST [52] was used to prepare the inoculums of the test organisms. Briefly, colonies picked from 24 h old cultures grown on nutrient agar plates were used to make suspensions of the test organisms in saline solution (0.85% NaCl) to give an optical density of approximately 0.1 at 600 nm. The suspension was then diluted a hundred-fold before use.

### 3.5. Antibacterial Susceptibility Test

The sensitivity of each crude extract of the plant was determined using the agar well diffusion method as described by [53], with modifications. The prepared bacterial suspension (100 μL) was inoculated into sterile molten Mueller-Hinton agar medium at 50 °C in a MacCarthney bottle, mixed gently and then poured into a sterile petri dish and allowed to solidify. A sterile 6 mm diameter cork borer was used to bore wells into the agar medium after which the wells were filled up with approximately 100 μL of 10 mg/mL extract solution taking care to prevent spillage onto the surface of the agar medium. The plates were then allowed to stand on the laboratory bench for 1 h to allow proper diffusion of the extract into the medium before incubation at 37 °C for 24 h, and thereafter the zones of inhibition were observed and measured. Ciprofloxacin (2 μg/mL) was used as a positive control, and distilled water was used as the negative control while 5% Dimethyl sulphoxide (DMSO) was also tested to determine its effect on each organism.

### 3.6. Determination of the Minimum Inhibitory Concentration (MIC) and Minimum Bactericidal Concentration (MBC)

The broth microdilution assay method of EUCAST [52] was used to determine the MICs for the susceptible *Listeria* isolates in sterile disposable flat-bottomed 96-well microtiter plates. Two-fold serial dilutions using sterile distilled water were carried out from 10 mg/mL stock plant extracts to make nine test concentrations ranging from 0.039 to 10 mg/mL for each solvent extract. Double strength Mueller-Hinton broth (100 μL) was introduced into all the 96 wells and 50 μL of the varying concentrations of the extracts were added in decreasing order along with 50 μL of the test organism suspension. Column 1 was used as the sterility wells containing 100 μL of sterile distilled water in addition to the 100 μL of Mueller-Hinton broth, column 2 was used as the positive control wells containing 100 μL of the broth, 50 μL of Ciprofloxacin and 50 μL of the test organism whilst column 3 was used as the negative control wells containing 100 μL of the broth, 50 μL sterile distilled water and 50 μL of the test organism whilst columns 4 to 12 were used as test wells containing 100 μL of the broth, 50 μL of the extract concentration and 50 μL of the test organism. The plates were then incubated at 37 °C for 18–24 h. Results were read visually by adding 40 μL of 0.2 mg/mL of ρ-iodonitrotetrazolium violet (INT) dissolved in sterile distilled water into each well [54]. A pinkish coloration is indicative of microbial growth because of their ability to convert INT to red formazan [55]. The MIC was recorded as the lowest concentration of the extract that prevented the appearance of visible growth of the organism after 24 h of incubation [52].

Sudjana *et al*.’s method [56] was used to determine the minimum bactericidal concentration (MBC) from the MIC broth microdilution assays by subculturing 10 μL volumes from each well that did not exhibit growth after 24 h of incubation and spot inoculating it onto fresh Mueller-Hinton agar plates. The plates were incubated for 48 h after which the numbers of viable colonies were counted. The MBC was defined as the lowest concentration killing more than or equal to 99.9% of the inoculum compared with initial viable counts [56].

### 3.7. Rate of Kill Assay

The time kill assay was done according to the method of Odenholt *et al*. [57] as described by Akinpelu *et al*. [50]. The selected test *Listeria* isolates namely *L. ivanovii* (LEL 18), *L. grayi* (LAL 15), *L. monocytogenes* (LAL 8) and *L. ivanovii* (LEL 30) were used for the rate of kill studies as representatives of the *Listeria* species used in the study. The turbidity of the 18 h old test *Listeria* was first standardized to 10^8^ cfu/mL. Four different concentrations of the plant extract were made starting from the MIC to 4× MIC value for each test organism. A 0.5 mL volume of known cell density from each organism suspension was added to 4.5 mL of different concentrations of the extracts solutions, held at room temperature and the rate of kill determined over a period of 2 h. Exactly 0.5 mL volume of each suspension was withdrawn at 15 min intervals and transferred to 4.5 mL of nutrient broth recovery medium containing 3% “Tween 80” to neutralize the effects of the antimicrobial compound carryovers on the test organisms [50]. The suspension was then serially diluted and 0.5 mL was plated out for viable counts using the pour plate method. The plates were thereafter incubated at 37 °C for 48 h. The control plates contained the test organism without the plant extracts. The emergent colonies were counted and compared with the counts of the culture control.

### 3.8. Statistical Analysis

SPSS 19.0 version for Windows program (SPSS, Inc.) at a 95% confidence level was used to determine the one way ANOVA, means and standard deviations.

## 4. Conclusions

This study revealed the anti-*Listerial* activities of both the crude *n*-hexane and aqueous extracts of *Garcinia kola* seeds with the *n*-hexane extracts being more active and bactericidal. Further studies to determine the extracts interactions with standard antibiotics and to also isolate and characterize the active principles in the *n*-hexane extract are subjects of on-going investigation in our group. We conclude that *Garcinia kola* seeds hold promise as a potential source of therapeutic compounds that can be exploited in effective anti-*Listerial* therapy.

## Figures and Tables

**Table 1:** The anti-*Listerial* activities of Ciprofloxacin and the crude *n*-hexane and aqueous extracts of *Garcinia kola* seeds.

ORGANISM	N-H	A	C	ORGANISM	N-H	A	C
*L. grayi* (LAL 13)	0	0	20 ± 3.055	*L. ivanovii* (LEL 18)	9 ± 1	8 ± 0	20 ± 3.215
*L. ivanovii* (LEL 17)	8 ± 0.577	0	19 ± 1.528	*L. ivanovii* (LEL 29)	0	0	8 ± 0.577
*L. ivanovii* (LEL 30)	17 ± 0.577	0	30 ± 0.577	*L. ivanovii* (LEL 15)	0	0	13 ± 2.082
*L. ivanovii* (LDB 11)	9 ± 0.577	0	20 ± 1	*L. ivanovii* (LDB 9)	9 ± 1	0	25 ± 2.082
*L. ivanovii* (LEL9)	9 ± 0.577	0	16 ± 2.082	*L. ivanovii* (LDB 10)	13 ± 0	8 ± 0.577	25 ± 0.577
*L. ivanovii* (LEL 1)	16 ± 1.155	11 ± 1	17 ± 0.577	*L. ivanovii* (LEL 2)	0	0	28 ± 1.528
*L. ivanovii* (LEL 5)	0	0	11 ± 0.577	*L. ivanovii* (LEL 6)	0	0	11 ± 1.732
*L. ivanovii* (LEL 3)	0	0	35 ± 3.055	*L. ivanovii* (LEL 4)	0	0	14 ± 1
*L. ivanovii* (LEL 19)	0	0	25 ± 4.041	*L. ivanovii* (LEL 10)	0	0	20 ± 2.082
*L. ivanovii* (LAL 9)	11 ± 0.577	0	25 ± 1.732	*L. ivanovii* (LAL 11)	10 ± 0.577	8 ± 0	17 ± 2.646
*L. grayi* (LAL 12)	8 ± 0	0	17 ± 1.155	*L. ivanovii* (LAL 10)	10 ± 5.77	8 ± 0.577	15 ± 2.082
*L. grayi* (LAL 15)	10 ± 2.082	8 ± 0	18 ± 2.082	*L. ivanovii* (LAL 14)	0	0	30 ± 2.517
*L. ivanovii* (LDB 1)	0	0	15 ± 2.082	*L. ivanovii* (LDB 2)	0	0	14 ± 0
*L. ivanovii* (LAL 6)	0	0	19 ± 1.155	*L. ivanovii* (LAL5)	0	0	20 ± 1.528
*L. ivanovii* (LAL 7)	0	0	20 ± 1.528	*L. monocytogenes* (LAL 8)	13 ± 5.77	10 ± 1.155	12 ± 1
*L. ivanovii* (LDB 7)	17 ± 0.577	10 ± 0.577	27 ± 0.577	*L. ivanovii* (LDB 12)	16 ± 1.528	10 ± 0.577	25 ± 1.528
*L. ivanovii* (LDB 3)	11 ± 0	8 ± 0.577	15 ± 1	*L. ivanovii* (LDB 8)	0	0	20 ± 1.732
*L. ivanovii* (LEL 7)	0	0	9 ± 1	*L. ivanovii* (LEL 8)	0	0	30 ± 1.528
*L. ivanovii* (LEL 14)	0	0	35 ± 2	*L. ivanovii* (LEL 16)	12 ± 1	8 ± 0.577	15 ± 1.528
*L. grayi* (LAL 3)	0	0	13 ± 3.055	*L. ivanovii* (LAL 4)	0	0	20 ± 2
*L. ivanovii* (LAL 2)	13 ± 2.082	8 ± 0.577	16 ± 1	*L. ivanovii* (LAL 1)	0	0	20 ± 2

(number ± number) denotes mean of three replicates zone of inhibition diameter in mm± standard deviation in mm; N-H: denotes *n*-Hexane extract; A: denotes aqueous extract; C: denotes Ciprofloxacin.

**Table 2:** The Minimum Inhibitory Concentration (MIC) and Minimum Bactericidal Concentration (MBC) of crude *n*-hexane and aqueous extracts of *Garcinia kola* seeds against susceptible *Listeria* isolates.

Organism	Extracts			

	*n*-Hexane		Aqueous	

	MIC (mg/mL)	MBC (mg/mL)	MIC (mg/mL)	MBC (mg/mL)
*L. ivanovii* (LEL9)	0.079	10	_	_
*L. ivanovii* (LEL 18)	0.079	10	10	>10
*L. ivanovii* (LAL 10)	0.157	10	10	>10
*L. ivanovii* (LEL 30)	0.157	0.625	_	_
*L. ivanovii* (LEL 16)	0.157	10	10	>10
*L. monocytogenes* (LAL 8)	0.079	5	10	>10
*L. ivanovii* (LDB 12)	0.157	5	>10	>10
*L. ivanovii* (LDB 10)	0.079	10	10	>10
*L. ivanovii* (LEL 1)	0.157	10	>10	>10
*L. ivanovii* (LAL 11)	0.625	10	10	>10
*L. ivanovii* (LDB 3)	0.079	10	10	>10
*L. grayi* (LAL 15)	0.079	10	10	>10
*L. grayi* (LAL 12)	0.625	10	_	_
*L. ivanovii* (LDB 11)	0.079	10	_	_
*L. ivanovii* (LAL 2)	0.157	10	10	>10
*L. ivanovii* (LEL 17)	0.625	10	_	_
*L. ivanovii* (LDB 7)	0.079	10	10	>10
*L. ivanovii* (LDB 9)	0.625	5	_	_
*L. ivanovii* (LAL 9)	0.079	10	_	_

Key: _ denotes not determined
